# SP-D Serum Levels Reveal Distinct Epithelial Damage in Direct Human ARDS

**DOI:** 10.3390/jcm10040737

**Published:** 2021-02-12

**Authors:** Konrad Peukert, Benjamin Seeliger, Mario Fox, Caroline Feuerborn, Andrea Sauer, Patrick Schuss, Matthias Schneider, Sascha David, Tobias Welte, Christian Putensen, Christoph Wilhelm, Folkert Steinhagen, Christian Bode

**Affiliations:** 1 Department of Anesthesiology and Intensive Care Medicine, University Hospital Bonn, Venusberg-Campus 1, 53127 Bonn, Germany; konrad.peukert@ukbonn.de (K.P.); Mario.Fox@ukbonn.de (M.F.); Caroline.Feuerborn@ukbonn.de (C.F.); Andrea.Sauer@ukbonn.de (A.S.); christian.putensen@ukbonn.de (C.P.); f.steinhagen@vk.shg-kliniken.de (F.S.); 2 Department of Respiratory Medicine and German Centre of Lung Research (DZL), Hannover Medical School, Carl-Neuberg-Str. 1, 30635 Hannover, Germany; seeliger.benjamin@mh-hannover.de (B.S.); welte.tobias@mh-hannover.de (T.W.); 3 Department of Neurosurgery, University Hospital Bonn, Venusberg-Campus 1, 53127 Bonn, Germany; Patrick.schuss@ukbonn.de (P.S.); matthias.schneider@ukbonn.de (M.S.); 4 Institute for Intensive Care Medicine, University Hospital Zurich, Raemisstr, 100, 8091 Zurich, Switzerland; sascha.david@usz.ch; 5 Department of Nephrology and Hypertension, Hannover Medical School, Carl-Neuberg-str. 1, 30635 Hannover, Germany; 6 Institute of Clinical Chemistry and Clinical Pharmacology, University Hospital Bonn, Venusberg-Campus 1, 53127 Bonn, Germany; cwilhelm@uni-bonn.de

**Keywords:** phenotype, inflammation, sepsis, lung injury, respiratory distress syndrome, angiopoietins, pneumonia, cytokines, interleukin-6, precision medicine

## Abstract

Acute respiratory distress syndrome (ARDS) is a heterogeneous syndrome with multiple underlying diseases. Particularly epithelial damage results from direct (e.g., pneumonia) rather than indirect lung injury (e.g., nonpulmonary sepsis), which is more likely associated with endothelial damage. Hence, targeting ARDS patients based on their molecular phenotypes is a promising approach to improve outcome. With regard to distinct inflammatory responses and subsequent lung damage in direct ARDS due to the causing pathogen, we quantified markers of epithelial and endothelial damage and pro-inflammatory cytokines in patients with ARDS triggered by bacterial, viral, and atypical pathogen pneumonia or indirect ARDS. The serum levels of interleukin-6 (IL-6) and interleukin-8 (IL-8), lung epithelial injury markers surfactant protein D (SP-D), and soluble receptor for advanced glycation end-products (sRAGE) as well as endothelial injury marker angiopoietin-2 (Ang-2) from 49 patients with distinct types of ARDS were analyzed by multiplex immunoassay. Epithelial damage marker SP-D was significantly higher in direct ARDS caused by viral and atypical pathogens in contrast to ARDS caused by typical bacterial pneumonia and nonpulmonary sepsis. In contrast, sRAGE levels did not differ due to the causing pathogen. Patients with atypical pathogen pneumonia related ARDS showed significantly lower Ang-2 levels compared to patients with viral and indirect ARDS. Patients with viral and atypical pneumonia related ARDS possessed significantly lower serum IL-6 levels compared to bacterial pneumonia related ARDS and IL-6 levels in atypical pneumonia related ARDS were significantly lower than in indirect ARDS. Current findings report a potential difference in ARDS biomarkers due to the underlying disease and pathogen.

## 1. Introduction

Acute respiratory distress syndrome (ARDS) is a heterogeneous syndrome with various underlying diseases. It is characterized by severe pulmonary inflammation that originates from either direct (e.g., pneumonia) or indirect (e.g., nonpulmonary sepsis) injury to the lung with subsequent endothelial and epithelial damage [1,2]. This heterogeneity between ARDS patients has most likely contributed to the failure of new therapies for ARDS in clinical trials, despite encouraging preclinical data [3]. Therefore, it is necessary to understand the biological differences among ARDS patients to tailor future clinical trials to distinct ARDS subphenotypes that respond better to a specific therapy. Most clinical studies of novel treatments for ARDS that targeted to the pulmonary epithelium or endothelium included a cohort of patients with various sources of pneumonia and indirect forms of ARDS [4]. Increasing evidence suggests that the magnitude of epithelial and endothelial injury differs between ARDS subphenotypes [1]. Angiopoietin-2 (Ang-2), which is a key activator of endothelial cells and increases vascular permeability, is highly expressed in serum of patients with indirect ARDS, suggesting that endothelial damage is more pronounced [1]. In contrast, surfactant protein-D (SP-D) and soluble receptor for advanced glycation end-products (sRAGE) have been identified as promising biomarkers to quantify epithelial damage and are elevated in patients with direct ARDS [1,2,3,5,6,7]. Furthermore, high SP-D and sRAGE serum concentrations were found to be associated with unfavorable outcomes in direct ARDS [5,6,7]. These previous studies pretend biological homology within patients with direct ARDS. However, this subtype is mainly triggered by pneumonia that is caused by various pathogens including viruses (e.g., influenza) as well as typical and atypical bacteria such as *Streptococcus pneumoniae* and mycoplasma pneumoniae, respectively. The inflammatory response and alveolar cell interaction towards these different classes of microbial pathogens differs significantly [8]. In particular, the pro-inflammatory cytokines IL-6 and IL-8 are released in response to pathogen recognition and are closely linked to the extent of pulmonary injury in ARDS [9,10]. We hypothesize that direct ARDS displays distinct lung damage and inflammatory response depending on the pathogens involved. Therefore, the current study investigates markers of both epithelial and endothelial damage as well as inflammation in patients with direct ARDS caused by bacterial, viral, or atypical pneumonia in comparison with indirect ARDS triggered by nonpulmonary sepsis.

## 2. Material and Methods

### 2.1. Patients

ARDS was diagnosed according to the Berlin Definition [11]. Bronchoalveolar lavage fluid (BALF) was collected via direct bronchoscopy for bacterial and virological testing within 24 h of ICU admission. Based on the resulting pathogen (Figure S1), together with medical history and radiological reports, patients were categorized in viral (*n* = 19), bacterial (*n* = 15) and atypical pneumonia (*n* = 5) related ARDS. Individuals with nonpulmonary sepsis were categorized as having indirect ARDS (*n* = 10). Patients were recruited at University Hospital Bonn (viral, bacterial, atypical, and indirect) and Hannover Medical School (viral). 

### 2.2. Biomarker Measurement

Serum obtained from ARDS patients was collected at both hospitals in a Serum-Gel Monovette (S-Monovette, Sarstedt AG & Co. KG, Nuembrecht, Germany) within 24 h after ICU admission. Samples were centrifuged at 2500 G for 10 min at room temperature. Immediately afterwards, the samples were stored at −80 °C until further processing. Levels of lung epithelial injury markers SP-D and RAGE, endothelial injury marker Ang-2 as well as pro-inflammatory cytokines IL-6 and IL-8 were analyzed by multiplex immunoassay (Luminex Assay, Bio-Techne, Minneapolis, MN, USA). 

### 2.3. Statistics

Statistical analysis was performed using R Version 4.0.3 (Vienna, Austria). Patient characteristics were compared by Kruskall–Wallis or Fishers exact test and expressed as median, 25%, and 75% percentile. In nonparametric data between group comparison after a significant Kruskall–Wallis test was performed using a Dunns test with Benjamini–Hochberg adjustment for multiple comparison. For better comparability and to achieve normal-distribution, biomarker data was Log-transformed and is presented as an individual value with mean ± SD and distribution shown as violin plot. *p*-values of pairwise *t*-tests with Benjamini–Hochberg adjustment are shown if *p*-value of global ANOVA was <0.05.

## 3. Results

### 3.1. Patient Characteristics

Patient characteristics were compared, stratified by viral, bacterial, atypical pneumonia related ARDS and indirect ARDS (Table 1). While no significant differences in demographics, immunocompromised conditions, disease severity and ventilator settings, and the percentage of community acquired pneumonia were observed, the procalcitonin (PCT) serum levels were significantly different between groups, with higher levels in bacterial and indirect ARDS (Table 1, Kruskal–Wallis *p* = 0.024).

### 3.2. Epithelial Damage Differs between Different Etiologies of ARDS

**Figure 1:**
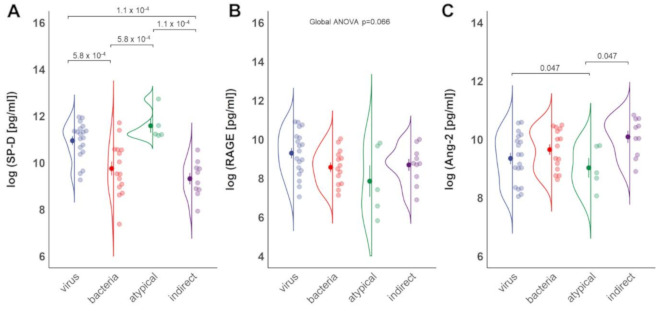
Serum SP-D and Ang-2 levels differ in patients with different ARDS sub-phenotypes. Blood samples of patients with ARDS were obtained and analyzed via multiplex immunoassay. Patients with ARDS caused by viral (*n* = 19), bacterial (*n* = 15), and atypical pathogen pneumonia (*n* = 5) were included. The group of indirect ARDS comprises 10 patients. (**A**,**B**) SP-D and RAGE concentrations in patients with ARDS of viral, bacterial, and atypical pathogen geneses and indirect ARDS. (**C**) Ang-2 levels in the same patient groups. Mean ± SD of log-transformed data is shown, pairwise *t*-tests with Benjamini–Hochberg adjustment after global ANOVA.

To test whether the degree of lung epithelial injury in distinct subtypes of pneumonia related ARDS differs based on the causing pathogen, we analyzed serum concentrations of SP-D and sRAGE in patients with viral, bacterial, and atypical pneumonia and patients with nonpulmonary sepsis. As shown in Figure 1A, SP-D serum levels of patients with viral and atypical pneumonia were significantly increased compared to individuals with bacterial pneumonia (*p* < 0.001). Surprisingly, SP-D levels did not differ in ARDS patients caused by bacterial pneumonia and indirect lung injury while there was a significant difference between viral as well as atypical pneumonia ARDS when compared to indirect ARDS (Figure 1A, *p* < 0.001). In contrast, no differences in serum sRAGE levels between patients with pneumonia and indirect ARDS were detected (Figure 1B). We next examined endothelial damage by monitoring Ang-2 serum levels. Here, patients with atypical pathogen pneumonia related ARDS showed significantly lower Ang-2 levels compared to patients with viral and indirect ARDS (Figure 1C, *p* = 0.047).

### 3.3. Inflammation Differs between Sub-Groups of ARDS

**Figure 2:**
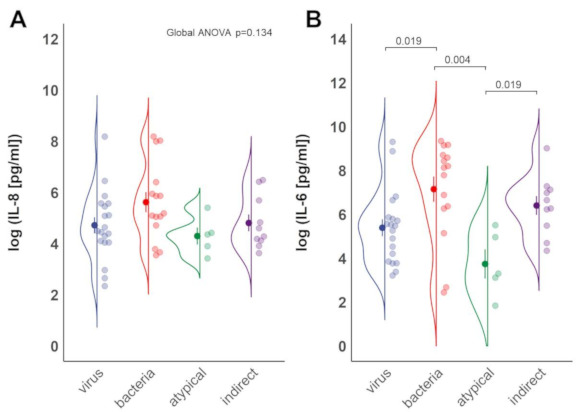
IL-6 and IL-8 serum concentrations in patients with different ARDS sub-phenotypes. Blood samples of patients classified as described in Figure 1 were analyzed for (**A**) IL-8 and (**B**) IL-6 concentrations. Mean ± SD of log-transformed data is shown, pairwise *t*-tests with Benjamini–Hochberg adjustment after global ANOVA.

The inflammatory cytokines IL-6 and IL-8 are released after epithelial injury in the lungs and elevated serum levels are a molecular hallmark of ARDS [9,10,12]. While no differences were observed between groups for IL-8 levels (Figure 2A), patients with bacterial pneumonia as well as indirect ARDS exhibited significantly elevated IL-6 levels compared to individuals with atypical pneumonia (Figure 2B, *p* = 0.004 and *p* = 0.019, respectively). Further, significant, higher IL-6 levels were found in patients with bacterial pneumonia ARDS compared to viral pneumonia related ARDS (Figure 2B, *p* = 0.019).

## 4. Discussion

In the current study, the epithelial damage marker SP-D was the highest in patients with viral and atypical pneumonia related ARDS, while sRAGE levels did not differ significantly between groups (Figure 1A,B). Ang-2 levels significantly differed between virus and atypical pneumonia related ARDS and were found elevated in individuals with indirect ARDS (Figure 1C). Patients with atypical pneumonia related ARDS demonstrated significantly reduced serum levels of IL-6 compared to bacterial pneumonia related or indirect ARDS. ARDS by viral pneumonia was characterized by lower serum IL-6 levels than bacterial pneumonia related ARDS (Figure 2B).

To our knowledge, the present study shows for the first time that levels of the epithelial injury, marker SP-D significantly differ depending on the causing pathogen of direct ARDS. Our data suggest that ARDS induced by atypical and viral pneumonia is associated with pronounced lung epithelial damage compared to bacterial pneumonia related ARDS. Consistent with this, marked variability of serum SP-D concentration due to different microorganisms was currently found in patients with community-acquired pneumonia [13,14]. Here, patients with atypical pathogen pneumonia exhibited significantly higher serum SP-D concentrations than patients with pneumonia caused by typical bacteria like *Streptococcus pneumonia* or *Haemophilus influenza* [13]. Furthermore, increased serum SP-D concentrations were found in patients with A/H1N1 virus related pneumonia compared to healthy controls [7]. Pronounced epithelial damage in viral rather than bacterial pneumonia related ARDS might be due to faster spreading of viral than bacterial infections, leading to a burst-like course of epithelial damage [15]. Alternatively, the intracellular pathology of both viruses as well as atypical pathogens causing intense desquamation, necrosis and cessation of mucocilial clearance in epithelial cells might be responsible for the more pronounced epithelial damage [15,16]. In contrast to our results, others found lower SP-D levels in patients with viral pneumonia compared to pneumonia either caused by intracellular or extracellular bacteria [14]. Since patients admitted to the ICU were excluded from that study and SP-D levels increase with the severity of pneumonia [14,17], the difference might be explained by less severity of illness in comparison to our patients. Yet, this remains unclear since the small sample sizes of this report prevented reasonable adjustment for illness severity in our patient groups. In contrast to SP-D, we monitored no differences in sRAGE levels between distinct groups of ARDS. The time-dependent release of both damage markers during lung injury might explain the observed differences between SP-D and sRAGE levels. Murine models demonstrated an initial elevation of serum SP-D levels up to 60 h after lung injury while sRAGE concentrations peaked already in the first 24 h of disease onset [18,19,20]. Since the current study investigated the serum levels of both epithelial damage markers only within 24 h of ICU admission, further studies that take the time-dependent release of sRAGE and SP-D into account are needed to confirm our results. 

Notably, we observed significantly reduced IL-6 concentrations in patients with atypical pathogen pneumonia ARDS compared to ARDS by bacterial pneumonia. This is confirmed in recent studies revealing lower IL-6 levels in atypical pathogen pneumonia than in pneumonia caused by typical bacterial infections, such as Streptococcus pneumonia. Variations in virulence, subverting pattern recognition receptor signaling or limited recognition of different molecular patterns of atypical bacteria through innate inflammatory pathways producing IL-6 and IL-8 might be responsible for the reduced inflammatory response [21]. At least atypical pathogens like *Mycoplasma* and *Legionella pneumoniae* are known to be able to alter the cytokine profile of human pulmonic epithelial cells by direct interference with signaling cascades responsible for IL-6 and IL-8 production [22].

Predictive enrichment strategies can profoundly improve clinical trials. Identifying patient characteristics to select those who are more likely to respond to an intervention pave the way for precision medicine approaches [1,3,23]. The consideration of specific molecular markers of lung epithelial and endothelial damage as well as inflammatory status might be essential for the success of specific therapies aiming at pulmonary injury or inflammation in ARDS, particularly because enrichment strategies are very useful to select patients in treatment trials in highly heterogenous syndromes such as ARDS [1,3,23,24]. For instance, patients with community-acquired pneumonia possessing high serum concentrations of pro-inflammatory cytokines including IL-6 and inappropriate low cortisol levels benefited from a therapy with dexamethasone in terms of mortality and ICU admission rates [25]. In ARDS, anti-inflammatory therapy tended to improve oxygenation and airway pressures but were not associated with a consistent survival benefit [26,27]. Many clinical studies that investigated specific therapies to attenuate epithelial damage or hyperinflammation in ARDS enrolled patients without considering distinct phenotypes of ARDS [27]. This could obscure treatment effects, which might only be apparent in subgroups preferentially accessible for treating epithelial injury, endothelial damage, or hyperinflammation. 

This study has several limitations. First, biomarkers measured in serum are surrogate parameters for the pulmonary damage and inflammation and do not fully reflect the complexity of pathological mechanisms that take place in the lungs. In addition, the current study investigated a limited number of biomarkers that cannot summarize the complexity of both the cellular damage and the immune response in ARDS. Even when measuring serum SP-D, sRAGE and IL-6 concentrations are well established for differentiation of distinct molecular phenotypes of ARDS [1,2,3]. Future studies should include additional biomarkers associated with lung epithelial injury (e.g., Clare Cell protein 16), endothelial damage (e.g., E-selectin), coagulation (e.g., protein C), and inflammation (e.g., IL-18) to further enhance molecular phenotyping [28].

Second, the current study found a variance in severity of the disease and outcome among the groups of patients included, which might limit the comparability. Even though these differences were not significant, small sample sizes of the current study prevented adjusting for confounders which could possibly be associated with different biomarker constellations. For instance, others who differentiated direct and indirect ARDS via differential expression of biomarkers found significant associations between biomarker levels and patients variables such as vasopressor use, race, and APACHE III scores [1]. Therefore, the current results can be understood as an indication of an altered pattern of expression in ARDS by different pathogens and underlying diseases, but should be evaluated in larger patient collectives adjusted for known and possible confounders by multivariate analysis to strengthen the validity of the findings.

Third, patients with viral pneumonia are susceptible for bacterial coinfections [29]. Even though there was no microbiologically-confirmed bacterial coinfection in the viral group of the current study, some patients with primary viral infection displayed relatively high IL-6 levels, which could indicate an undetected bacterial superinfection.

With regard to epithelial damage and systemic inflammation, current findings may report a potential for differentiation in ARDS biomarkers due to underlying disease and inducing pathogens. This heterogeneity within subtypes of direct ARDS should be considered in future clinical studies investigating treatments which are specifically targeted at pulmonary epithelial injury and inflammation.

## Figures and Tables

**Table 1:** Characteristics of patients with acute respiratory distress syndrome (ARDS) caused by viral, bacterial or atypical pathogen pneumonia and indirect ARDS.

Characteristic	Virus (*n* = 19)		Bacteria (*n* = 15)		Atypical Pathogens (*n* = 5)		Indirect (*n* = 10)		*p*
Age (y)	56.5	(48–63)	59	(37–70)	67	(61–71)	50	(43–55)	0.063
Male (%)	14	(74)	12	(80)	3	(60)	9	(90)	0.512
BMI (kg/m²)	32.6	(27.0–37.2)	27.7	(24.6–36.7)	26.1	(25–29.4)	28.3	(27.8–33.2)	0.301
Diabetes (%)	3	(16)	3	(20)	1	(20)	3	(30)	0.785
Immunosuppression (%)	5	(26)	2	(13.3)	1	(20)	1	(10)	0.824
Community acquired pneumonia (%)	19	(100)	11	(73)	5	(100)	N/A		0.061
Steroids (%)	4	(21)	5	(33)	1	(20)	6	(60)	0.164
PaO2/FiO2 ratio (mmHg)	72	(60–99)	86	(73–147)	95	(90–162)	129	(72–158)	0.131
PEEP (cmH2O)	19	(14–20)	18	(14–20)	18	(16–20)	17	(14–18)	0.723
Driving pressure (cmH2O)	9	(7–14)	9	(6–14)	10	(9–11)	10	(8–12)	0.946
Tidal volume (ml/kg predicted body weight)	3.5	(2.4–6.7)	2.7	(2–6.4)	1.9	(1.4–3.8)	4.1	(2.6–7)	0.389
Procalcitonin (PCT_ (µg/L)	1.9	(0.9–17.6)	27.2	(6.3–70.1)	0.4	(0.4–3.4)	11.3	(2.1–67.9)	0.024 *
Lactate (mmol/L)	1.4	(1.2–2.3)	2.8	(1.8–4.7)	1.3	(1.0–1.8)	1.6	(1.3–4.6)	0.170
SOFA score	10	(8–12)	8	(5–10)	8	(8–10)	9	(6–10)	0.131
ICU mortality (%)	6	(32)	5	(33)	3	(60)	3	(30)	0.673

Data are presented as median, 25%, and 75% percentile. Abbreviations: BMI = Body mass index; SOFA score = sepsis related organ failure assessment score (best assumed for CNS), ICU = intensive care unit, PEEP = positive end-expiratory pressure. Median, 25%, and 75% percentile is shown, Kruskall–Wallis or Fishers exact test. * Dunns test with Benjamini–Hochberg adjustment for multiple-group comparison for PCT after Kruskal–Wallis test (*p* = 0.024): Viral vs. Bacteria *p* = 0.0228; Bacteria vs. Atypical pathogens *p* = 0.031.

## Supplementary Materials

The following are available online at https://www.mdpi.com/2077-0383/10/4/737/s1, Figure S1: Results of microbial testing.
