# Supplementary material for: SP-D Serum Levels Reveal Distinct Epithelial Damage in Direct Human ARDS
Source: J Clin Med. 2021 Feb 12;10(4):737. doi: 10.3390/jcm10040737 (PMC7917979; doi:10.3390/jcm10040737)
Supplement: Supplementary file 1 [file jcm-10-00737-s001.pdf]

Virus (n = 19)	Bacteria (n = 15)	Atypical Pathogens (n = 5)	Indirect (n = 10)
Influenza A (n=16)	Klebsiella pneumoniae (n=2)	Legionella (n=1)	Enterococcus faecalis (n=1)
Human metapneumovirus (n=1)	Escherichia coli (n=2)	Clamydia pneumoniae (n=1)	Staphylococcus aureus (n=2)
Herpes simplex virus (n=1)	Klebsiella oxytoca(n=2)	Pneumocystis carinii (n=1)	Klebsiella pneumoniae (n=1)
Parainfluenza virus (n=1)	Streptococcus pneumoniae (n=2)	n/a (n=2)	n/a (n=6)
	Streptococcus pyogenes (n=1)		
	Staphylococcus aureus (n=2)		
	n/a (n=4)		
